# Beyond Traditional Risk Factors: Integrating Epicardial Adipose Tissue into the Comorbidity Landscape of HFpEF

**DOI:** 10.3390/jcm14176139

**Published:** 2025-08-30

**Authors:** Marius-Dragoș Mihăilă, Bogdan Caloian, Florina Iulia Frîngu, Diana Andrada Irimie, Ioan Alexandru Minciună, Dana Pop

**Affiliations:** 1 4th Department of Internal Medicine, Department of Cardiology Rehabilitation, “Iuliu Hațieganu” University of Medicine and Pharmacy, 400012 Cluj-Napoca, Romania; dragos_mihaila1998@yahoo.com (M.-D.M.); florina.fringu@elearn.umfcluj.ro (F.I.F.); gurzau.diana@elearn.umfcluj.ro (D.A.I.); minciuna.ioan.alexandru@elearn.umfcluj.ro (I.A.M.); pop67dana@gmail.com (D.P.); 2 Department of Cardiology Rehabilitation, Clinical Rehabilitation Hospital, 400437 Cluj-Napoca, Romania

**Keywords:** epicardial adipose tissue, heart failure with preserved ejection fraction, echocardiography, cardiac magnetic resonance imaging, SGLT2 inhibitors

## Abstract

Epicardial adipose tissue (EAT), the visceral fat layer next to the myocardium, has become an important focus in heart failure with preserved ejection fraction (HFpEF). When enlarged and inflamed, EAT increases pericardial restraint, releases fibroinflammatory mediators, and disrupts myocardial energetics, thereby reproducing the high-pressure, exercise-intolerant HFpEF phenotype regardless of body mass index. Modern echocardiography, cardiac CT, and MRI, enhanced by artificial intelligence texture analytics, now enable precise depot-specific quantification, making EAT a measurable therapeutic target. Early interventional studies suggest that caloric restriction, bariatric surgery, SGLT2 inhibitors, GLP-1 receptor agonists, statins, PCSK9 antibodies, and colchicine can reduce EAT volume or alter its inflammatory profile, with concurrent improvements in haemodynamics and biomarkers. However, definitive outcome trials are still pending. Priority directions include standardising imaging cut-offs, mapping EAT immune–metabolic niches, and testing combined metabolic–inflammatory regimens to translate EAT modulation into precision therapy for HFpEF. This review aims to synthesise current mechanistic, diagnostic, and therapeutic insights on EAT in HFpEF and outline future research priorities.

## 1. Introduction

Heart failure with preserved ejection fraction (HFpEF) now accounts for over 50% of all heart failure (HF) cases and still lacks a proven disease-modifying therapy (apart from SGLT2 inhibitors) [1,2]. Therefore, clinicians must focus on alleviating symptoms and identifying and managing comorbidities. Its incidence continues to rise in parallel with an ageing, increasingly obese, hypertensive population, emphasising the need to identify modifiable risk factors [3].

Among such drivers, epicardial adipose tissue (EAT), the fat layer situated between the visceral pericardium and the myocardium, has evolved from an anatomical curiosity to a potential pathogenic organ. Landmark echocardiographic studies initially validated EAT thickness as a surrogate for visceral adiposity [4], while more recent CT and CMR research emphasises its metabolic activity and spatial proximity to the coronary micro-circulation [5]. Unlike abdominal or subcutaneous depots, EAT shares the coronary blood supply, lacks a fascial barrier, and can, thus, influence cardiac structure and function through direct paracrine, vasocrine, and mechanical signalling.

Clinical evidence linking EAT to HFpEF is increasing. Invasive haemodynamic studies demonstrate that excess EAT amplifies pericardial restraint and elevates left atrial and pulmonary pressures during exercise [6]. Meta-analytic data confirm that greater EAT volume or thickness is associated with poorer diastolic function, higher NT-proBNP, and reduced peak VO_2_, hallmarks of HFpEF, independent of body mass index [7,8]. Large multicentre CMR cohorts further indicate that each 1 mL/m^2^ increase in indexed EAT predicts higher HF hospitalisation and mortality over 24 months [9].

Mechanistically, expanded EAT becomes pro-inflammatory, profibrotic, and mechano-active. It releases cytokines, adipokines, and micro-RNAs that contribute to myocardial fibrosis and microvascular dysfunction, while its volume limits ventricular filling, two factors that reflect the stiff-heart, high-pressure physiology seen in HFpEF [10]. Additionally, systems biology analyses indicate that neuro-humoral activation and metabolic inflammation within EAT are significant components of the obese–diabetic HFpEF phenotype [11].

Yet, important gaps persist. No investigation has demonstrated a direct causal chain from excess epicardial adipose tissue to the full HFpEF clinical syndrome [12]. Quantitative cut-offs that define “pathological” EAT still vary between echocardiography, CT, and MRI, and cross-modality agreement remains uncertain [13]. Moreover, although preliminary data suggest that SGLT-2 inhibitors, GLP-1 receptor agonists, and bariatric surgery can reduce or modulate EAT, it remains unclear whether these changes translate into lasting symptomatic relief or prognostic benefits for patients with HFpEF [14].

The aim of this narrative review is to provide an integrative synthesis of current knowledge on the role of EAT in HFpEF. Specifically, we aim to (1) summarise mechanistic insights into how EAT may contribute to HFpEF pathophysiology, (2) evaluate available imaging modalities for its assessment, (3) review current and emerging therapeutic strategies targeting EAT, and (4) outline key gaps and future research directions. By bringing together evidence from basic science, imaging studies, and clinical interventions, our goal is to offer a coherent framework that informs both clinical practice and future investigations in this evolving field.

## 2. Methods

This manuscript is a narrative review aimed at providing an integrative overview of the role of EAT in HFpEF, with emphasis on diagnosis, treatment, and future research directions. Topics and subsections were selected a priori to ensure comprehensive coverage from anatomical and physiological foundations through pathophysiological mechanisms to clinical diagnosis and therapeutic approaches. We searched MEDLINE/PubMed, Web of Science, and Embase for peer-reviewed literature using combinations of the following terms: “epicardial adipose tissue”, “EAT”, “heart failure with preserved ejection fraction”, “echocardiography”, “cardiac computed tomography”, “cardiac magnetic resonance imaging”, “diastolic dysfunction”, “SGLT2 inhibitors”, “GLP-1 receptor agonists”, “bariatric surgery”, and “anti-inflammatory therapy”. Where appropriate for each section, additional modifiers such as “anatomy”, “physiology”, “pathophysiology”, “diagnosis”, and “treatment” were applied. We prioritised studies most pertinent to adult HFpEF and identified additional articles by screening the reference lists of key publications. As a narrative review, we synthesised the evidence to maximise breadth, clinical relevance, and clarity.

## 3. EAT in HFpEF vs. HFrEF

Since 2010, when the role of EAT in HF became increasingly evident and studied, significant differences have been found between its effects on patients with HFpEF and those with reduced ejection fraction. Some studies have demonstrated that the EAT mass indexed to body surface area is lower in patients with HFrEF compared to healthy subjects in a control group [15]. In contrast, patients with HFpEF have higher amounts of epicardial adipose tissue compared to the control group, supporting the existence of a distinct, obesity-related phenotype of HFpEF [16], particularly in light of evidence showing that EAT is frequently associated with hypertension [17], obesity [18], type 2 diabetes mellitus [19], and atrial fibrillation [20], all of which are common comorbidities in patients with this type of HF. However, few studies directly compare patients with HFpEF to those with reduced ejection fraction regarding the amount and distribution of EAT. In one such study [21], it was found that the amount of EAT, evaluated by echocardiography, was significantly higher in patients with HFpEF and was correlated with older age, smaller left ventricular internal diameters, LVEDV index, and lateral and septal e’ values, correlations that were absent in patients with HFrEF.

Moreover, studies indicate that a greater amount of EAT is linked to improved cardiac function in patients with HFrEF [22], whereas in patients with HFpEF, it is associated with a more precarious cardiorespiratory status and increased mortality [23]. A possible explanation for these findings could be that EAT, functioning similarly to brown adipose tissue, acts as an energy reservoir; consequently, in reduced quantities, it correlates with an increased catabolic status, which is frequently observed in patients with HFrEF, and severe systolic dysfunction [24]. On the other hand, a recent study [12] found that a higher amount of EAT is an independent risk factor for endothelial dysfunction in patients with HFrEF, but not in those with preserved ejection fraction, even after adjusting for confounding factors such as dyslipidaemia, hypertension, obesity, and diabetes. In patients with HFrEF, it was also linked to increased peripheral arterial stiffness, which predicts adverse cardiovascular outcomes [25]. Conversely, recent studies focused on HFpEF have shown that EAT is present in greater quantities in women [26], increasing the likelihood of impaired exercise capacity in this demographic [6]. However, the link between sex, EAT, its effects, and HFpEF remains contentious, as some studies suggest that the impact of EAT on cardiovascular outcomes in HFpEF is independent of sex [27].

Although a growing body of research highlights significantly different associations and potential pathophysiological roles of EAT in HFpEF compared to HFrEF, the notable absence of direct comparisons and mechanistic investigations makes more comprehensive and focused studies necessary to clarify these divergent clinical impacts and guide phenotype-specific therapeutic strategies.

## 4. Pathophysiology of EAT in HFpEF

It is well known that obesity increases the risk of cardiovascular mortality and predisposes to the onset and progression of HF [28], both by increasing insulin resistance, promoting systemic inflammation, and increasing oxidative stress [29] and by its association with other cardiovascular risk factors such as dyslipidemia, diabetes mellitus, and hypertension [30]. It has also been found that obesity is more common in patients with HFpEF than in those with reduced ejection fraction [31]. Thus, an obesity-related phenotype of HFpEF has emerged that is also closely related to the presence of atrial fibrillation, chronic obstructive pulmonary disease, and obstructive sleep apnoea syndrome [29]. In clinical practice, anthropometric indices such as body mass index and abdominal circumference are the most commonly used to diagnose obesity [32]. However, recent studies have shown that these measurements do not correlate with cardiovascular risk [33], as they fail to distinguish between lean and adipose mass and do not provide information regarding the distribution of adiposity among individuals [34]. In contrast, waist-to-hip ratio and waist-to-height ratio have been demonstrated to correlate with increased cardiovascular risk [33], as they more accurately reflect visceral adiposity, which serves as a better predictor of cardiovascular risk than subcutaneous adiposity [34].

One such type of visceral adipose tissue (VAT) is EAT, which is particularly relevant compared to other categories of VAT in the development of HF, given that it is located beneath the visceral pericardium and in close proximity to the myocardium, without being separated by fascia [35]. Additionally, it shares the same vascularisation as the myocardial tissue, being supplied by branches of the coronary arteries [36]. It is also essential to distinguish between epicardial and pericardial adipose tissue, as they differ in terms of anatomical location and vascularisation, with the pericardial adipose tissue being vascularised by branches of the internal mammary artery [37], and also embryologically, with the EAT arising from the splanchnopleural mesoderm and the pericardial tissue originating from the primitive thoracic mesenchyme [38].

EAT plays several physiological roles. Firstly, because it is in direct contact with the myocardium and branches of the coronary arteries, it provides mechanical support and protection against deformation [39]. Secondly, it is believed to play a role in thermoregulation, as research shows that EAT expresses increased levels of UCP1 [10], a mitochondrial membrane protein involved in adaptive thermogenesis by shifting mitochondrial metabolism towards heat production at the expense of ATP [40], which is particularly characteristic of brown adipose tissue [41]. Last but not least, EAT acts as a reservoir of free fatty acids, which serve as the primary myocardial energy source under physiological conditions [42]. It has been demonstrated to exhibit higher rates of fatty acid incorporation and lipogenesis compared to adipose tissue in other locations [43].

Although EAT plays multiple essential physiological roles, under certain conditions, such as ones that may result in its quantitative increase or the alteration of its homeostasis and metabolic processes [44], it can become a significant pathological factor in the development of HF.

**Figure 1:**
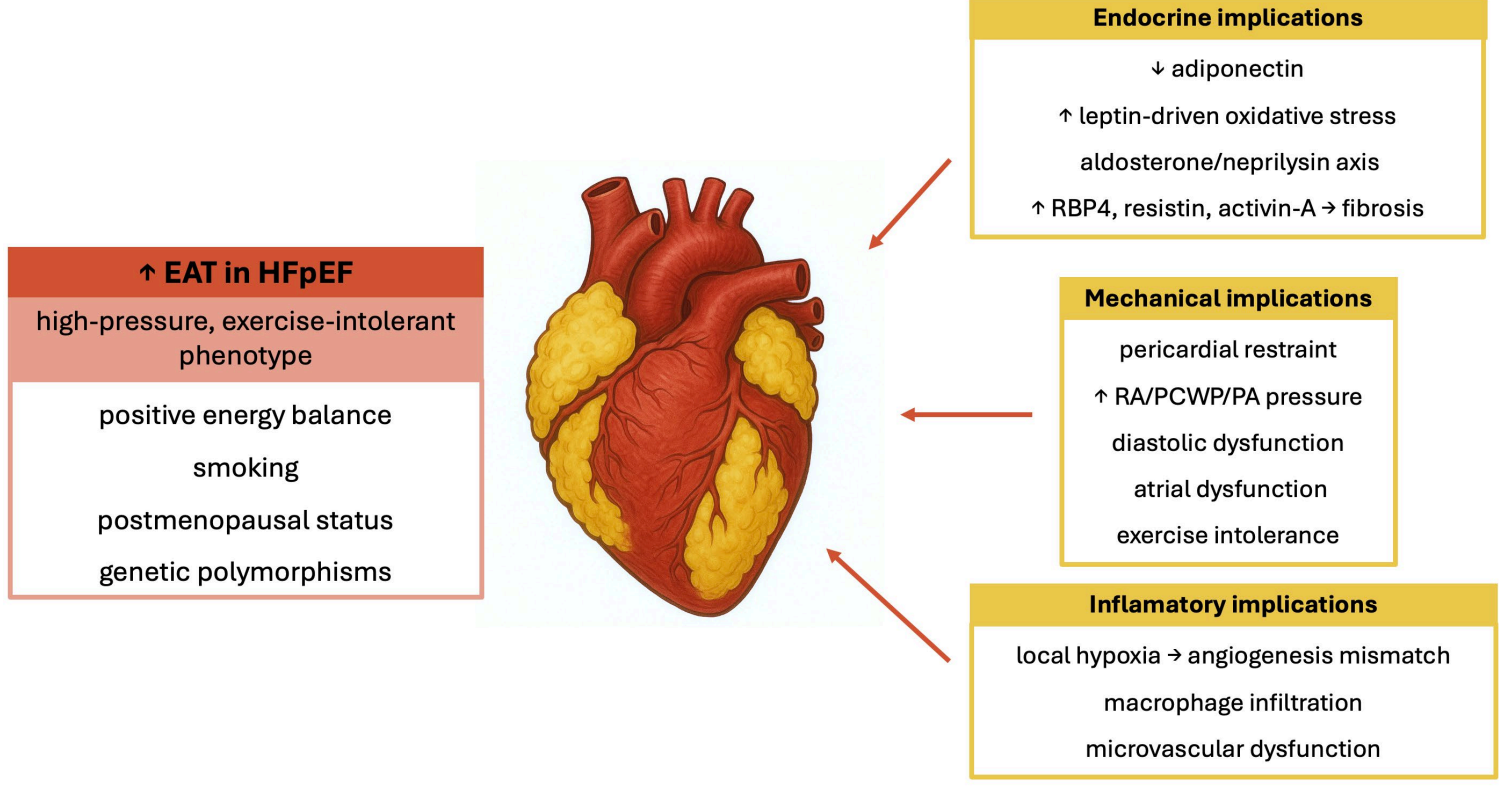
Mechanical, endocrine, and inflammatory implications of EAT in HFpEF. EAT, epicardial adipose tissue; HFpEF, heart failure with preserved ejection fraction; RBP4, retinol-binding protein 4; RA, right atrium; PCWP, pulmonary capillary wedge pressure; PA, pulmonary artery; References: [2,23,24,46,47,48,49,50,51,52,53,54,55,56,57].

The mechanisms by which EAT participates in the development and progression of HFpEF are currently a highly researched topic (Figure 1). Factors linked to increased EAT include, among others, a positive energy balance, smoking, postmenopausal status, and various genetic polymorphisms [2]. A potential explanation for changes in EAT metabolism is the theory of adipose tissue hypoxia. This theory proposes that as adipose tissue accumulates, the increased oxygen demand is not met by a corresponding rise in oxygen supply [45], along with alterations in angiogenesis in obese patients [46]. This theory indirectly links EAT to HFpEF by suggesting that inadequate oxygen supply to expanding EAT promotes a dysfunctional metabolic profile, which can impair myocardial relaxation and contribute to diastolic dysfunction.

### 4.1. Mechanical Effects of EAT

Under normal circumstances, the pericardium exerts compressive forces on the heart, supporting the coupling of left and right ventricular preload to sustain a relatively constant stroke volume despite variations in venous return. Like any cardiac chamber, the pericardium shows a curvilinear pressure–volume relationship, initially flat before rising steeply when stretched toward the steep part of its pressure–volume curve [58]. In individuals with significant obesity, cardiomegaly, and increased epicardial fat volume, the pericardial sac exhibits a steeper pressure–volume relationship, causing enhanced diastolic ventricular interaction and pericardial restraint [47]. This results in an exaggerated compressive force on the heart, leading to increased left- and right-sided filling pressures. Patients with HFpEF and elevated EAT show higher right atrial (RA) pressure, pulmonary capillary wedge pressure (PCWP), and pulmonary artery (PA) pressure at rest and during exercise. These changes occur independently of BMI, indicating a specific role for EAT beyond generalised obesity [59]. Mechanistically, this hemodynamic profile shows evidence of ventricular interdependence and square root signs on invasive right ventrivular (RV) pressure tracings [48], a hemodynamic marker suggestive of a constrictive physiology that is classically associated with constrictive pericarditis, but can also occur in other conditions, particularly restrictive cardiomyopathy [60]. The presence of such signs has been associated with EAT thickness, especially in women, indicating a sex-specific vulnerability to EAT-induced pericardial constraint [48]. Notably, these constrictive effects appear distinct from restrictive cardiomyopathy, as myocardial relaxation is typically preserved or even improved (as confirmed by echocardiography through increased septal e′ velocities), suggesting that the pericardium, not the myocardium, is the limiting factor in many HFpEF patients with excess EAT [61].

Regarding effects on atrial mechanical function, one of the earliest and most consistent associations identified is between EAT and left atrial (LA) mechanical dysfunction, a key contributor to atrial fibrillation in HFpEF. Cardiac magnetic resonance (CMR)-based studies have demonstrated that increased EAT volume is associated with reduced LA reservoir strain and larger LA volumes, independent of body mass index (BMI) [49]. This indicates that EAT may exert direct mechanical effects on atrial function regardless of overall obesity levels. It further suggests that EAT might locally influence atrial tissue, causing adverse remodelling [62].

Patients with elevated EAT adjacent to the left atrium (LA-EAT) commonly exhibit electrical remodelling, as shown in electrophysiological studies revealing prolonged atrial conduction times and large low-voltage areas, all of which promote arrhythmogenic substrate development [63], reflecting the combined effects of local inflammation and mechanical compression, which impair atrial conduction and promote ectopic activity [64]. LA-EAT may cause asynchronous atrial contraction by impairing regional deformation and diastolic function. This dyssynchrony promotes atrial fibrillation by supporting re-entrant circuit formation, raising atrial pressure, and worsening atrial enlargement [65]. Imaging studies, such as strain analysis, highlight this phenomenon through segmental delays in time-to-peak strain (TTP), particularly in the posterior and inferior LA walls adjacent to EAT deposits. These regions show flattened or delayed strain curves, indicating localised dyssynchrony that correlates with the extent and distribution of EAT [66].

EAT accumulation is consistently associated with reduced aerobic capacity, with multiple studies showing inverse relationships between EAT thickness and peak VO_2_, as well as arteriovenous oxygen difference (A-VO_2_diff), especially in HFpEF patients [23]. These associations remain significant after adjustment for BMI and waist circumference, highlighting the impact of EAT on both central and peripheral determinants of exercise intolerance [47]. In HFpEF, this is likely due to a combination of elevated filling pressures, impaired ventricular compliance, increased pulmonary congestion, and reduced peripheral oxygen extraction. These functional impairments are not solely caused by excess body weight, but are driven by altered microvascular function in both the central and peripheral territories, as well as cardiomyocyte function, possibly secondary to EAT-derived inflammatory mediators [50]. In contrast, HFrEF patients with lower EAT levels may experience exercise intolerance due to cardiac cachexia and muscle wasting, emphasising the contrasting prognostic implications of EAT in HFpEF vs. HFrEF [67].

### 4.2. Metabolic and Endocrine Effects of EAT

EAT is a distinct visceral fat deposit located directly on the myocardial surface without any intervening fascia, allowing for direct interactions between adipocytes and cardiomyocytes via a shared coronary microcirculation. This close anatomical and vascular connection promotes metabolic and paracrine crosstalk between EAT and the heart, making EAT a key regulator of local myocardial metabolism and endocrine signalling. Physiologically, EAT plays several vital roles. Metabolically, EAT releases a range of bioactive molecules, including adipokines and cytokines, which positively influence endothelial function and heart tissue health. Among these, adiponectin is essential, providing anti-inflammatory, anti-oxidative, anti-fibrotic, and anti-atherogenic benefits by improving endothelial nitric oxide availability and lowering oxidative stress and pro-inflammatory mediators such as interleukin 6 and C-reactive protein [24,51]. Additional adipokines such as adrenomedullin and omentin enhance these protective effects by promoting vasodilation and maintaining endothelial homeostasis [68]. However, in pathological states such as obesity and metabolic syndrome (conditions frequently associated with HFpEF), the physiological functions of EAT are significantly disrupted [24]. Excessive expansion of EAT causes local hypoxia due to insufficient microvascular supply, triggering a phenotypic shift from a protective to a pro-inflammatory adipose tissue phenotype [24].

A recent study [52] has shown that rats with metabolic syndrome exhibit increased visceral fat accumulation, including EAT, along with an imbalance in adipokine secretion and a significant rise in leptin levels. These rats also show myocardial injury, mainly affecting the subepicardial myocardium, and the extent of this damage is specifically linked to leptin from EAT rather than circulating serum leptin. Leptin produced by EAT in metabolic syndrome fosters mitochondrial oxidative stress and dysfunction, initiates apoptosis via mitochondrial pathways, and diminishes cardiomyoblast cell viability through activation of the PKC/NADPH oxidase/reactive oxygen species (ROS) pathway. Additionally, this EAT-derived leptin stimulates inflammation in cardiomyocytes by promoting AP-1 nuclear translocation through the same signalling cascade. Leptin also promotes the secretion of aldosterone and angiotensin II, resulting in elevated neprilysin levels, which promote sodium retention and volume overload [69]. This has led to the proposal of the leptin–aldosterone–neprilysin axis as a new mechanism through which EAT dysfunction may contribute to the development of HF, alongside the downregulation of thermogenic genes such as peroxisome proliferator-activated receptor gamma (PPAR-γ) [53].

Furthermore, levels of serine proteinase inhibitor A3, a regulator of inflammation, are significantly elevated in the EAT of HF patients compared to those without HF [70]. Studies comparing EAT to subcutaneous adipose tissue (SAT) show a notable upregulation of p53 mRNA in EAT, a factor known to promote reactive oxygen species production, adipocyte apoptosis, and local inflammation, thereby contributing to coronary atherosclerosis and myocardial remodelling [71].

EAT expansion leads to increased secretion of several adipokines, with significant effects on myocardial structure and function. Retinol-binding protein 4 (RBP4), elevated in obesity and HFpEF [54], is produced by EAT and drives a harmful cycle of insulin resistance and inflammation through activation of the TLR-4 pathway, fostering ROS production, cardiomyocyte hypertrophy, and reduced glucose uptake, thereby impairing cardiac metabolism and efficiency [68]. Resistin, another adipokine released by EAT, links inflammation and cardiovascular disease by promoting endothelial dysfunction and atherogenesis via NF-κB-mediated upregulation of adhesion molecules, although its specific role in HFpEF remains underexplored [55]. Omentin-1, an anti-inflammatory and antioxidant adipokine highly expressed in healthy EAT, decreases with obesity [72]. This reduction is linked to insulin resistance, increased inflammation, atherosclerosis, and poor outcomes in heart failure, positioning omentin-1 as a potential therapeutic target for reducing inflammation in obesity-related HFpEF [73]. Activin-A, another EAT-derived adipokine, promotes myocardial fibrosis by stimulating collagen accumulation through the ALK4 receptor, contributing to structural remodelling and impaired diastolic relaxation [74]. In HFpEF, this mechanism may worsen lipotoxicity and elevate oxidative stress, thereby deteriorating diastolic dysfunction. Activin-A also disrupts insulin signalling in cardiomyocytes, increasing insulin resistance and contractile dysfunction [56]. Together, these adipokines emphasise the diverse endocrine functions of EAT in regulating inflammation, oxidative stress, metabolism, and fibrosis, thereby affecting myocardial remodelling and dysfunction in pathological states.

Although multiple studies have demonstrated links between EAT expansion and HFpEF, establishing a direct causal relationship remains difficult. Research on EAT in relation to HFpEF is relatively novel, and current evidence is still preliminary, with definitive proof from large, prospective clinical trials lacking. Furthermore, as highlighted in the European Society of Cardiology Guidelines, HFpEF is a multifactorial condition where various comorbidities interact cumulatively to affect both disease development and progression. EAT likely contributes to HFpEF pathophysiology as part of this broader network of risk factors, rather than acting as a sole cause.

## 5. Imaging-Based Evaluation of EAT

### 5.1. Transthotacic Echocardiography

Two-dimensional echocardiography allows for visualisation of EAT as the echo-free (or, in large volumes, hyperechoic) space between the outer myocardial wall and the visceral pericardium [75]. Standard parasternal long-axis and midventricular short-axis views are employed to align the ultrasound beam perpendicular to anatomical landmarks, such as the aortic annulus in the long-axis view or the papillary muscle level in the short-axis view, and to measure maximal fat thickness on the right ventricular free wall at end-systole over three cardiac cycles [76]. End-systolic measurement is preferred because epicardial fat gets compressed during diastole, which can underestimate thickness [76].

Multiple population-based studies report excellent interobserver and intraobserver agreement for EAT thickness measurement [77]. However, moderate agreement between echocardiographic and multidetector CT measurements has been observed, particularly when end-diastolic values are used, highlighting the importance of standardised end-systolic protocols [78]. With values reported between 1 and 23 mm, echocardiographic EAT thickness varies widely, reflecting differences in visceral fat distribution among individuals [76]. Median values of approximately 7 mm in men and 6.5 mm in women have been observed in unselected clinical cohorts [77]. Proposed diagnostic cut-offs include ≥9.5 mm for metabolic syndrome in men (≥7.5 mm in women) [77] and ≥7 mm to identify subclinical coronary artery disease [79], although these thresholds may need adjustment across different ethnic groups.

A recent study [80] demonstrated that echocardiographic assessment of EAT at the Rindfleisch fold, a pericardial recess situated between the right ventricle and the aorta, provides robust prognostic information in HF. Measurement at this site was proven to be highly reproducible and correlate closely with EAT thickness and volume determined by CMR imaging, as the ventricular curvature here maximises the space between pericardial layers, facilitating clear visualisation and accurate quantification [81]. In this study, among HF patients undergoing implantable cardioverter–defibrillator (ICD) placement for both primary and secondary prevention, each mm increase in EAT conferred a 16% higher risk of the composite endpoint (HF-related deaths, new hospital admissions for HF, and atrial and ventricular arrhythmic events) and a 14% greater risk of arrhythmic events, highlighting its strong prognostic utility.

### 5.2. Cardiac Magnetic Resonance Imaging

CMR imaging has become the reference technique for quantifying total body and visceral fat, especially EAT [82], as it provides excellent delineation of both visceral and parietal pericardial layers, enabling accurate volumetric measurement of EAT [83].

Several sequences are suitable for EAT assessment. Fast spin echo T1-weighted “black-blood” imaging reliably estimates EAT volume, although it is not yet standard in all CMR protocols [83]. More commonly, steady-state free-precession cine acquisitions, which are already integral to ventricular functional studies, provide accurate and reproducible pericardial fat quantification [84]. Volumetric analysis is usually performed at end-diastole: EAT is manually outlined on contiguous short-axis slices, slice areas are multiplied by slice thickness, and the resulting volumes are summed to determine total EAT burden [85]. Recent developments include fully automated, non-contrast algorithms that speed up analysis and enhance reproducibility [86].

CMR’s principal clinical advantage is its safety profile: the examination is non-invasive, free of ionising radiation, and routinely performed without contrast agents, an asset for patients with renal impairment or iodine allergy [85]. Limitations remain, notably restricted scanner availability, high operating costs, and the confined environment, which may preclude use in claustrophobic individuals or those with non-MR-compatible implants [84].

A recent prospective CMR study of 966 patients [84] identified epicardial-fat T1 time (EAT-T1) as an independent predictor of major adverse events, including HF hospitalisation, all-cause mortality, and non-fatal myocardial infarction, beyond traditional risk factors and ventricular function. Multivariable regression showed that only age and male sex were linked to longer EAT-T1, reflecting CT data that connect cardiac fat attenuation to these non-modifiable factors. A high EAT-T1 likely indicates fat tissue fibrosis and inflammation, features previously associated with adverse outcomes when evaluated through CT fat attenuation [87]. These abnormalities may convert EAT from an energy-rich, anti-inflammatory reservoir into a source of pro-inflammatory and pro-fibrotic mediators that contribute to coronary and myocardial dysfunction.

Another recent CMR study using deep learning segmentation [88] introduced a fully automated pipeline that extracts EAT directly from routine four-chamber cine MRI, providing a time-efficient surrogate for total EAT burden. Across 100 subjects, the four-chamber intrapericardial fat area correlated strongly with reference short-axis-derived volumes, confirming earlier observations that this 2D metric tracks true fat burden and is clinically linked to diastolic dysfunction, hypertension, insulin resistance severity [89], and non-alcoholic fatty liver disease [90]. By converting existing cine acquisitions into quantitative fat maps without additional sequences, this methodology enables large-scale retrospective or prospective studies and supports clinical workflows where comprehensive CT quantification is impractical [88].

### 5.3. Cardiac Computed Tomography

Contemporary cardiac-gated CT (cCT) enables high-fidelity visualisation of EAT, allowing both volumetric and thickness quantification. After manual delineation of the visceral pericardium and myocardial borders, voxels with attenuation between −30 and −190 HU are assigned to EAT [91]. Subsequent 3D rendering provides total and compartment-specific volumes (atrial, ventricular, and pericoronary). Beyond volume, cCT uniquely characterises tissue composition through the fat attenuation index (FAI), a continuous measure of mean EAT density that serves as a surrogate for adipocyte size and inflammatory status [92]. Moreover, contrast-enhanced coronary CT angiography (CCTA), which is now the guideline-endorsed first-line test for suspected coronary artery disease [93], has a unique ability to visualise both the arterial lumen and the immediately adjacent pericoronary fat (PCAT), making it an unparalleled tool for studying the EAT–artery interface [85].

Mechanistic experiments demonstrate that cytokines released by inflamed coronary segments inhibit preadipocyte maturation, generating small lipid-poor adipocytes with higher X-ray attenuation [94]. The resultant FAI elevation, therefore, serves as a non-invasive signature of vascular inflammation [95]. Elevated PCAT FAI independently predicts future acute coronary events and its prognostic power diminishes after initiation of anti-inflammatory therapies, highlighting both its pathophysiological specificity and modifiability [96]. AI-driven models combining FAI with plaque metrics further refine individual risk stratification [97].

A higher periatrial EAT (PAAT) volume and higher PAAT FAI are associated with incident atrial fibrillation, post-ablation recurrence, and persistence, independent of body mass index [98]. Importantly, the radial FAI gradient (high density near the atrial myocardium that normalises centrally) surpasses absolute FAI in predicting atrial fibrillation burden [99]. Likewise, significant heterogeneity in PAAT FAI has been identified as an independent predictor of atrial fibrillation relapse after pulmonary vein isolation [100].

From a practical perspective, integrating EAT imaging into routine HFpEF evaluation involves balancing diagnostic benefit with accessibility, cost, and patient suitability (Table 1). Transthoracic echocardiography is the most feasible option, as it is widely available, inexpensive, and already part of standard HFpEF assessments, allowing for quick, reproducible EAT thickness measurements without adding to patient burden [75,76,77,78,79,80,81]. CMR offers highly precise volumetric and tissue characterisation data, but its routine use is limited by availability, cost, examination duration, and contraindications such as claustrophobia or non-MR-compatible devices [82,83,84,85]. Cardiac CT and CCTA provide exceptional assessments of anatomy and tissue composition, and when performed for simultaneous purposes such as CAD evaluation, EAT quantification can be incorporated with minimal extra cost and time [85,91,92,93]. However, radiation exposure and contrast use are still important considerations. In clinical practice, the selection of the modality should, thus, be customised based on the diagnostic question, patient profile, and resource availability, with echocardiography serving as a practical first-line option and CT and CMR reserved for specific cases where detailed quantification or tissue characterisation could impact management.

## 6. Therapeutic Strategies Targeting EAT

### 6.1. Non-Pharmacological Interventions

Sustained lifestyle modification is an effective, non-invasive strategy for limiting EAT expansion [10]. Recent studies of severely obese adults showed that six months of caloric restriction combined with moderate aerobic exercise resulted in a 32% reduction in EAT thickness and concurrent regression of left-ventricular hypertrophy and diastolic dysfunction [101]. Additionally, it lowered circulating TNF-α, increased lipocalin concentrations, and improved myocardial strain parameters [102]. Even brief interventions seem beneficial: two weeks of continuous training in patients with type 2 diabetes mellitus decreased EAT volume and myocardial triglyceride content while enhancing aerobic capacity and insulin sensitivity [103].

Physical activity also seems essential in hypertensive populations. The first study to examine this relationship showed that higher habitual activity levels and better objective physical performance scores were inversely associated with EAT thickness, while daily sitting time had no independent link [103]. The inverse relationship is biologically plausible, as even modest activity can reduce visceral and ectopic fat deposits [104]. Conversely, physical inactivity may promote systemic inflammation and insulin resistance, mechanisms that impair muscle function and could account for the observed connection between poorer performance and thicker EAT [105].

Bariatric (metabolic) surgery has an even more significant impact, with multiple studies documenting volumetric EAT reductions in the months following surgery [10] and one series reporting a 27% decrease at six-month follow-up [106]. Among obese patients with HFpEF, both lifestyle changes and metabolic surgery improve micro- and macrovascular endothelial dysfunction, favourably remodel the left ventricle, enhance diastolic performance, and boost exercise tolerance and quality of life [10]. Whether these morphological improvements lead to better long-term outcomes, however, still needs to be confirmed in prospective trials.

Taken together, evidence from both interventional and observational studies indicates that weight loss strategies, whether behavioural or surgical, can significantly reduce EAT and simultaneously enhance cardiac structure, function, and metabolic health (Table 2). Regular physical activity and maintaining physical performance appear especially important in preventing EAT accumulation. Robust outcome-oriented trials are still needed to determine whether actively modifying EAT results in tangible reductions in cardiovascular morbidity and mortality.

### 6.2. Lipid-Lowering Therapies

Beyond their lipid-centric benefits, statins exert a broad spectrum of cholesterol-independent (pleiotropic) actions. Experimental and clinical data show that statins enhance endothelial nitric oxide bioavailability, attenuate oxidative stress, stabilise atherosclerotic plaques, modulate immune and inflammatory pathways, inhibit vascular smooth muscle proliferation, and confer antithrombotic properties [107]. Additional observations indicate favourable effects on bone metabolism and a possible reduction in dementia risk [107]. Considering these various pleiotropic benefits, researchers have focused on examining whether statins could similarly reduce EAT.

Early clinical studies demonstrated that atorvastatin and high-intensity statin regimens consistently reduced EAT thickness and volume across diverse populations, such as patients with aortic stenosis [108], recent percutaneous coronary intervention [109], and atrial fibrillation [110], and that the effect was more pronounced with higher doses or more potent molecules compared to moderate-intensity alternatives such as pravastatin or simvastatin/ezetimibe [109]. Subsequent research confirmed that these anatomical changes are accompanied by reduced secretion of EAT-derived inflammatory mediators and decreased activation of the local inflammasome, an effect observed in vitro in cultured EAT adipocytes [111]. Importantly, subcutaneous fat does not display similar changes, emphasising the depot-specific action of statins [111].

Furthermore, reduced CT attenuation of EAT after statin therapy may indicate decreased inflammation or regression of the perivascular vasa vasorum [112], whereas experimental data suggest that statins downregulate UCP1 and other thermogenic genes in brown adipocytes, possibly reducing brown adipose tissue activity within the pericoronary compartment, which could be an alternative mechanism for lowering radiological density [113].

Reliable assessment of these qualitative and quantitative changes now benefits from specialised, highly precise segmentation software, which shows excellent agreement with manual ground truth in both non-contrast calcium score and contrast cCT data sets [114]. Because statins also improve endothelial function and suppress systemic inflammatory cytokines, automated EAT analysis may enhance overall cardiovascular risk evaluation beyond traditional lipid metrics [114].

A recent meta-analysis synthesising diverse imaging studies confirms a statistically significant, though modest, statin-related reduction in EAT, an effect that appears to be dose-dependent and not fully explained by LDL-cholesterol lowering alone [115]. These findings reinforce the idea that atherosclerosis involves both intraluminal and perivascular pathology and position EAT as a viable therapeutic target [115]. Standardised imaging protocols, mechanistic studies on adipose tissue phenotypes, and trials designed to detect major endpoints are needed to clarify whether modulating EAT through statins or future pharmacological agents results in additional cardiovascular benefits.

Recent evidence suggests that pro-protein convertase subtilisin/kexin type-9 (PCSK9) plays a key role at the intersection of EAT biology and cardiovascular risk. Recent studies have shown that EAT can synthesise and secrete PCSK9, with local expression closely associated with the extent of adipose inflammation, yet it remains largely unrelated to circulating PCSK9 levels [116]. Since the chemokine and cytokine profile linked to PCSK9 in EAT reflects pathways involved in monocyte recruitment and NF-κB activation, the data indicate a feed-forward loop where inflammation increases PCSK9, which then further enhances inflammatory signalling within the fat tissue and potentially in the nearby myocardium and coronary wall [116].

Therapeutic modulation of the PCSK9 axis is also increasingly promising. In a prospective cohort of 24 dyslipidaemic patients treated for six months with either evolocumab or alirocumab, transthoracic echocardiography showed a 20.39% reduction in EAT thickness [117]. This anatomical change occurred regardless of the extent of LDL-cholesterol reduction and was similar between the two monoclonal antibodies [117]. This finding suggests that PCSK9 inhibition can reduce EAT independently of its lipid-lowering effect, possibly by disrupting the inflammatory crosstalk described earlier.

### 6.3. Anti-Inflammatory Therapies

Targeting inflammation is widely regarded as the intuitive approach for treating patients with HFpEF who exhibit a maladaptive EAT phenotype. However, this strategy has never been tested with endpoints focused specifically on EAT itself [118]. Experience with interleukin-1 blockade demonstrates both its promise and its pitfalls: in a proof-of-concept study, the IL-1 receptor antagonist anakinra reduced systemic inflammatory markers and temporarily increased peak aerobic capacity, but a subsequent phase II trial failed to replicate these functional improvements [118].

Inflammation originating within EAT has emerged as an important, potentially modifiable substrate for atrial fibrillation, another key comorbidity in HFpEF. In a prospective clinical series of 122 patients undergoing catheter ablation, low-dose colchicine (0.5 mg daily for two weeks) did not influence early (three-month) outcomes, but it nearly halved mid-term atrial fibrillation recurrence at 12 months in those with an LA EAT volume above the cohort median of 22.4 cm^3^ (10.5 vs. 34.2%, *p* = 0.06). No benefit was observed when EAT burden was smaller [119]. These findings imply that the anti-inflammatory effects of colchicine become clinically significant only when the adipose inflammatory reservoir is substantial. Experimental work supports and extends this concept. In a mouse model of postoperative atrial fibrillation caused by lipopolysaccharide-induced acute inflammation [57], EAT-derived cytokines, neutrophil and macrophage infiltration, and a higher neutrophil-to-lymphocyte ratio created a pro-arrhythmic environment. These changes were associated with QTc prolongation, Ca^2+^-handling abnormalities, and increased atrial fibrillation inducibility [57]. Colchicine administered once at an intermediate dose (0.10–0.40 mg kg^−1^; human-equivalent ≈ 0.5 mg) normalised immune cell profiles, shortened QTc, stabilised the myocardial microtubule network, and reduced arrhythmia episodes, whereas lower or higher doses were ineffective or toxic [57]. Overall, the clinical and pre-clinical data show that EAT-driven inflammation increases the risk of early atrial fibrillation recurrence and postoperative atrial fibrillation, and that colchicine can reduce this risk if EAT volume is significant and dosing is carefully adjusted.

### 6.4. Anti-Hyperglycaemic Therapies

EAT promotes insulin resistance, oxidative stress, local inflammation, and fibrosis, all of which are linked to diastolic dysfunction and poorer haemodynamics in HFpEF [14]. Sodium–glucose co-transporter-2 inhibitors (SGLT2is) consistently reduce this depot: meta-analyses show that EAT volume or thickness decreases even when overall body mass index remains unchanged [120], an effect accompanied by higher circulating adiponectin and lower leptin [121]. Clinical imaging studies confirm the signal but show drug-specific heterogeneity. Empagliflozin reduced EAT volume in one six-month trial but caused only non-significant changes in two shorter studies, whereas dapagliflozin, canagliflozin, ipragliflozin, and luseogliflozin each induced meaningful reductions over 12 to 24 weeks. Meanwhile, in vitro, dapagliflozin also improved epicardial adipocyte differentiation [122]. These changes are believed to result from a drug-induced shift towards fat oxidation: chronic glycosuria mimics fasting, reduces insulin, promotes free-fatty-acid β-oxidation, and increases ketone production, thereby enhancing “fat burning” and improving adipocyte insulin sensitivity [123]. Consistent with this paradigm, SGLT2is normalise the lipogenesis-to-lipolysis ratio, curb lipid peroxidation, dampen HMG-CoA-reductase activity, and reduce both visceral and ectopic liver fat more than subcutaneous fat [122]. Anti-inflammatory benefits mirror these metabolic effects. Meta-analysis reveals notable increases in adiponectin and decreases in interleukin-6 and TNF-receptor-1, along with enhanced HOMA-IR [124]. In vivo, a four-week course of dapagliflozin reduced EAT glucose uptake by 21.6% and increased myocardial flow reserve by 30%, findings consistent with decreased epicardial inflammation and restored paracrine support of the coronary microcirculation [123]. Overall, roughly one-fifth reductions in EAT thickness or volume have now been reproducibly documented across several trials and imaging modalities [123].

Overall, the evidence suggests that SGLT2is exert depot-specific, glucose-independent fat-oxidative, anti-lipogenic, and anti-inflammatory effects that alter epicardial fat. By relieving EAT-driven mechanical constraints, lowering local cytokine spill-over, and enhancing coronary microvascular function, these changes may partially explain the drugs’ established cardiovascular benefits, although definitive mechanistic studies are still required.

Accumulating evidence suggests that drugs targeting the glucagon-like peptide-1 (GLP-1) pathway act directly on EAT rather than simply reducing it through overall weight loss. Human EAT expresses functional GLP-1 receptors and, as recently shown, also glucose-dependent insulinotropic polypeptide (GIP) and glucagon receptors, mainly on macrophages and subsets of adipocytes [125]. The receptor transcript profile correlates with genes that promote β-oxidation and browning while inhibiting lipogenesis, suggesting that incretin or dual/triple agonists could reprogramme EAT metabolism at its core [125].

Multiple small trials demonstrate that GLP-1 mimetics (exenatide, liraglutide, semaglutide, and dulaglutide) and even the DPP-4 inhibitor sitagliptin reduce EAT volume or thickness within 12–26 weeks in individuals with type 2 diabetes: most agents exhibit a clear dose-dependent response, although high-dose liraglutide (1.8 mg daily) was neutral in two studies while lower doses (1.2 mg) were effective [126]. Reported mechanisms include weight loss related fat redistribution, enhanced β-oxidation, the suppression of adipogenesis [127], and improved systemic metabolic indices (HbA1c, triglycerides, and waist circumference) [128].

The STEP-HFpEF trial linked semaglutide to improved symptoms, lower NT-proBNP/CRP levels, and fewer adjudicated events in obese HFpEF, supporting the idea that EAT modulation, not weight loss alone, underpins cardiovascular benefit [129].

HFpEF is characterised by excess, inflamed EAT that stiffens the ventricle, hence, GLP-1 receptor activation may reverse this by reducing adipogenesis and inflammatory cytokine release [130]. In contrast, HFrEF is often associated with epicardial fat depletion (a putative marker of cachexia), so further EAT loss could be undesirable. Observational data indeed suggest limited or even adverse outcomes with GLP-1 receptor agonists in advanced HFrEF, illustrating the “epicardial fat paradox” [130].

Discovering GIP and glucagon receptors in EAT broadens the therapeutic target range and supports ongoing trials of dual GIPR/GLP1R and triple GLP1R/GIPR/GCGR agonists [125]. Clarifying receptor-specific signalling in EAT and categorising patients by HF type and epicardial fat load will be crucial to fully realise the cardiometabolic potential of incretin-based therapies.

**Table 2:** Therapeutic strategies targeting EAT.

Non-Pharmacological Interventions		Pharmacological Interventions		
Lifestyle Changes	Bariatric Surgery	Lipid-Lowering	Anti-Inflammatory	Anti-Hyperglycaemic
Caloric restriction + aerobic exercise 6 months→32% ↓ EAT thickness [101] ↑ aerobic capacity and insulin sensitivity [103]	Improves [10]: Endothelial function Diastolic performance Exercise tolerance Quality of life	Statins→dose-dependent ↓ EAT & local inflammation [115] PCSK9i→~20% ↓ EAT thickness independent of LDL [117]	IL-1RA→transient ↑ peak aerobic capacity [118] Colchicine→~50% ↓ mid-term AF recurrence in high LA-EAT patients [119]	SGLT2i→ ↓ EAT volume [120], ↑ adiponectin [124], ↑ MFR [123] GLP-1A→ ↓ EAT mass [126], improved symptoms and biomarkers [127]

EAT, epicardial adipose tissue; PCSK9i, pro-protein convertase subtilisin/kexin type-9 inhibitors; IL-1RA, interleukin 1 receptor antagonists; AF, atrial fibrillation; LA-EAT, left atrial epicardial adipose tissue; SGLT2is, sodium-glucose cotransporter 2 inhibitors; MFR; myocardial flow reserve; GLP-1As, glucagon-like peptide-1agonists.

The concept of therapeutically targeting EAT in HF is supported by growing evidence from lifestyle, surgical, and pharmacological interventions showing that reducing EAT can lead to improvements in cardiac structure, diastolic function, inflammatory status, and metabolic health, especially in HFpEF [10,101,102,103,104,105,106,107,108,109,110,111,112,113,114,115,120,121,122,123,124,125,126,127,128,129,130]. These findings suggest EAT may be a modifiable factor in disease progression. However, uncertainties persist. Data on long-term, definitive cardiovascular outcomes are limited, and in specific contexts like HFrEF, where reduced EAT may indicate adverse metabolic conditions or cachexia, further depletion could be harmful [130]. Therefore, therapeutic approaches should be personalised, based on HF phenotype, baseline EAT burden, and overall metabolic profile, and confirmed by outcome-driven prospective studies.

## 7. Future Directions in EAT Research

Emerging research is redefining EAT as a key, adjustable factor in HFpEF. Upcoming mechanistic studies will employ single-cell and spatial transcriptomics to map how distinct EAT immune–adipocyte niches interact with the coronary microcirculation and contribute to ventricular stiffening. This agenda, outlined in recent state-of-the-art reviews, describes EAT as “the missing link” in HFpEF pathophysiology [131].

Parallel advances in imaging promise significantly improved diagnostics: fully automated deep learning pipelines for CT and CMR now quantify EAT volume, fat attenuation index, and even T1/T2 texture within seconds, offering new prognostic markers that will be incorporated into prospective HFpEF trials and routine risk stratification workflows [132].

On the therapeutic front, investigators are now broadening the range beyond semaglutide to agents that simultaneously remodel epicardial fat and improve HFpEF physiology. Anti-inflammatory colchicine has entered a prospective randomised trial explicitly designed for HFpEF (COLpEF), aiming to determine whether reducing sterile pericardial inflammation shrinks EAT and enhances diastolic reserve [133]. On the neurohormonal axis, the non-steroidal mineralocorticoid receptor antagonist finerenone has demonstrated favourable remodelling of visceral fat, increased β-oxidation, and decreased oxidative stress in preclinical models, prompting interest in its potential to regress EAT in upcoming HFpEF imaging substudies [134]. Lifestyle and rehabilitation strategies are also gaining traction: high-adherence Mediterranean diet programmes correlate with smaller EAT depots independent of BMI, suggesting that diet quality, not just caloric balance, influences cardiac fat health [135], while structured aerobic–resistance training regimens have been shown to reduce EAT volume and improve peak VO_2_, positioning cardiac rehabilitation as a combined therapy for fitness and fat remodelling in HFpEF [136].

Moreover, adipose tissue has become an essential source of regenerative cells for cardiovascular repair [137]. Among these, adipose-derived mesenchymal stem cells (ASCs) can be easily expanded ex vivo, directed towards cardiomyocyte or endothelial phenotypes, and are an abundant source of angiogenic and immunomodulatory paracrine factors. Therefore, stem cells harvested from EAT, along with their secretome, show particular promise for myocardial infarction therapy because their native molecular profile is already suited to cardiac healing needs [137].

Together, these threads outline a research agenda that combines high-resolution EAT phenotyping with targeted metabolic or anti-inflammatory therapies to personalise HFpEF management.

## 8. Conclusions

EAT has shifted from being an anatomical bystander to a plausible driver of the HFpEF phenotype. Through invasive haemodynamics, advanced imaging, and molecular studies, excess and inflamed EAT correlates with the following three interacting pathways: pericardial and mechanical constraint, microvascular and cardiomyocyte inflammation, and endocrine–metabolic dysregulation. These pathways, together, reproduce elevated filling pressures, atrial dysfunction, and exercise intolerance. Modern echocardiography, CT, and CMR enable depot-specific quantification. Emerging texture/attenuation metrics (e.g., FAI) and parametric measures (e.g., T1/T2), combined with automated segmentation, help to move EAT from a risk marker towards a measurable therapeutic target. Interventions that alter weight and metabolism (caloric restriction and bariatric surgery), glucose–lipid pathways (SGLT2 inhibitors, GLP-1-based agents, statins, and PCSK9 inhibitors), and inflammation (e.g., colchicine) consistently reduce or remodel EAT and are linked with improvements in haemodynamics, biomarkers, and patient-centred outcomes. Nonetheless, the role of EAT in HFpEF remains a promising, though still developing, target of investigation. Further research is necessary to establish causality, standardise diagnostic thresholds, and confirm whether modifying EAT can lead to improved long-term outcomes.

## Figures and Tables

**Table 1:** Comparative table of EAT imaging modalities.

Imaging Modality	Diagnostic Accuracy	Reproducibility	Prognostic Value	Practical Feasibility
Transthoracic Echocardiography (TTE)	Visualises EAT as an echo-free space between the outer myocardial wall and visceral pericardium; optimal accuracy with end-systolic measurement [75,76]. Moderate agreement with CT when end-diastolic values used [78].	Excellent interobserver and intraobserver agreement [77]	EAT thickness at Rindfleisch fold correlates with CMR measurements and independently predicts HF-related death, HF admissions, and arrhythmic events [80,81]	Widely available, low cost, no radiation; already integrated into HFpEF assessments; quick acquisition
Cardiac Magnetic Resonance (CMR)	Gold standard for quantifying visceral fat; excellent delineation of pericardial layers; cine steady-state free-precession acquisitions provide accurate, reproducible volumes [82,83,84,85].	Highly reproducible, especially with volumetric analysis; automated algorithms enhance consistency [86].	EAT-T1 independently predicts HF hospitalisation, all-cause mortality, and MI beyond traditional risk factors [84,87]. Four-chamber cine-derived EAT area correlates with diastolic dysfunction and metabolic risk [88,89,90].	No radiation, non-invasive; limited by scanner availability, cost, exam time, and contraindications [84,85].
Cardiac Computed Tomography (cCT/CCTA)	High-fidelity visualisation of EAT; enables volumetric and thickness quantification; ability to measure FAI as surrogate for inflammation [91,92].	High reproducibility with manual/automated methods; allows 3D rendering of EAT [91].	Elevated pericoronary FAI predicts acute coronary events and is modifiable by anti-inflammatory therapy [95,96]; PAAT volume/FAI predicts atrial fibrillation incidence and recurrence [98,99,100].	Excellent anatomical detail; can be integrated into CCTA for CAD evaluation with minimal added time; limited by radiation and contrast exposure [85,91,92,93].

TTE, transthoracic echocardiography; EAT, epicardial adipose tissue; CMR, cardiac magnetic resonance; HF, heart failure; HFpEF, heart failure with preserved ejection fraction; CT, computed tomography; MI, myocardial infarction; cCT, cardiac-gated computed tomography; CCTA, coronary CT angiography; FAI, fat attenuation index; PAAT, periatrial adipose tissue; CAD, coronary artery disease.

## Data Availability

No data were created or analysed in this study.

## Abbreviations

The following abbreviations are used in this manuscript:

EAT	Epicardial adipose tissue
HFpEF	Heart failure with preserved ejection fraction
HF	Heart failure
SGLT2	Sodium–glucose cotransporter 2
CT	Computed tomography
MRI	Magnetic resonance imaging
NT-proBNP	N-terminal pro-B-type natriuretic peptide
GLP-1	Glucagon-like peptide-1
HFrEF	Heart failure with reduced ejection fraction
LVEDV	Left ventricular end-diastolic volume
VAT	Visceral adipose tissue
UCP1	Uncoupling Protein 1
ATP	Adenosine triphosphate
RA	Right atrium
PCWP	Pulmonary capillary wedge pressure
PA	Pulmonary artery
BMI	Body mass index
RV	Right ventricle
LA	Left atrium
CMR	Cardiac magnetic resonance
PKC	Protein Kinase C
NADPH	Nicotinamide Adenine Dinucleotide Phosphate
ROS	Reactive oxygen species
PPAR-γ	Peroxisome proliferator-activated receptor gamma
SAT	Subcutaneous adipose tissue
RBP4	Retinol-binding protein 4
TLR-4	Toll-like receptor 4
NF-κB	Nuclear Factor kappa B
ICD	Implantable cardioverter–defibrillator
cCT	Cardiac-gated computed tomography
FAI	Fat attenuation index
CCTA	Coronary CT angiography
PCAT	Pericoronary adipose tissue
PAAT	Periatrial adipose tissue
TNF-α	Tumor Necrosis Factor alpha
PCSK9	Pro-protein convertase subtilisin/kexin type-9
GIP	Glucose-dependent insulinotropic polypeptide
DPP-4	Dipeptidyl peptidase-4
CRP	C-reactive protein
GCG	Glucagon gene
ASCs	Adipose-derived mesenchymal stem cells
